# A New Epigenetic Mechanism of Temozolomide Action in Glioma Cells

**DOI:** 10.1371/journal.pone.0136669

**Published:** 2015-08-26

**Authors:** Anna-Maria Barciszewska, Dorota Gurda, Paweł Głodowicz, Stanisław Nowak, Mirosława Z Naskręt-Barciszewska

**Affiliations:** 1 Department of Neurosurgery and Neurotraumatology, Karol Marcinkowski University of Medical Sciences, Przybyszewskiego 49, 60–355, Poznan, Poland; 2 Institute of Bioorganic Chemistry of the Polish Academy of Sciences, Noskowskiego 12, 61–704, Poznan, Poland; University of Navarra, SPAIN

## Abstract

Temozolomide (TMZ) is an oral alkylating chemotherapeutic agent that prolongs the survival of patients with glioblastoma (GBM). Despite that high TMZ potential, progression of disease and recurrence are still observed. Therefore a better understanding of the mechanism of action of this drug is necessary and may allow more durable benefit from its anti-glioma properties. Using nucleotide post-labelling method and separation on thin-layer chromatography we measured of global changes of 5-methylcytosine (m^5^C) in DNA of glioma cells treated with TMZ. Although m^5^C is not a product of TMZ methylation reaction of DNA, we analysed the effects of the drug action on different glioma cell lines through global changes at the level of the DNA main epigenetic mark. The first effect of TMZ action we observed is DNA hypermethylation followed by global demethylation. Therefore an increase of DNA methylation and down regulation of some genes expression can be ascribed to activation of DNA methyltransferases (DNMTs). On the other hand hypomethylation is induced by oxidative stress and causes uncontrolled expression of pathologic protein genes. The results of brain tumours treatment with TMZ suggest the new mechanism of modulation epigenetic marker in cancer cells. A high TMZ concentration induced a significant increase of m^5^C content in DNA in the short time, but a low TMZ concentration at longer time hypomethylation is observed for whole range of TMZ concentrations. Therefore TMZ administration with low doses of the drug and short time should be considered as optimal therapy.

## Introduction

Malignant gliomas are the most prevalent type of primary brain tumour in adults. They constitute approximately 50% of all central nervous system tumors [1]. Glioblastoma multiforme (GBM) is the most lethal subtype with a mean patient survival of 8–12 months from time of diagnosis [2,3]. The conventional therapy for GBM includes surgery followed by radiotherapy and chemotherapy [4]. DNA alkylation reagents are the oldest class of anti-cancer drugs. They are currently in use, and remain important for the treatment of various types of cancers including brain tumours [5,6]. Alkylating agents damage DNA by formation of different small or bulky adducts with the nucleic acid bases. The most promising therapeutically active agent for brain tumors is temozolomide [7,8]. Temozolomide (TMZ) is an oral alkylating agent that is regarded effective and prolongs survival when administered during and after radiotherapy. Temozolomide interferes with the development of cancer cells, slowing down their growth and spread in the body. It is used as a first-line treatment for glioblastoma. TMZ also shows significant activity against recurrent glioma [9].

**Fig 1:**
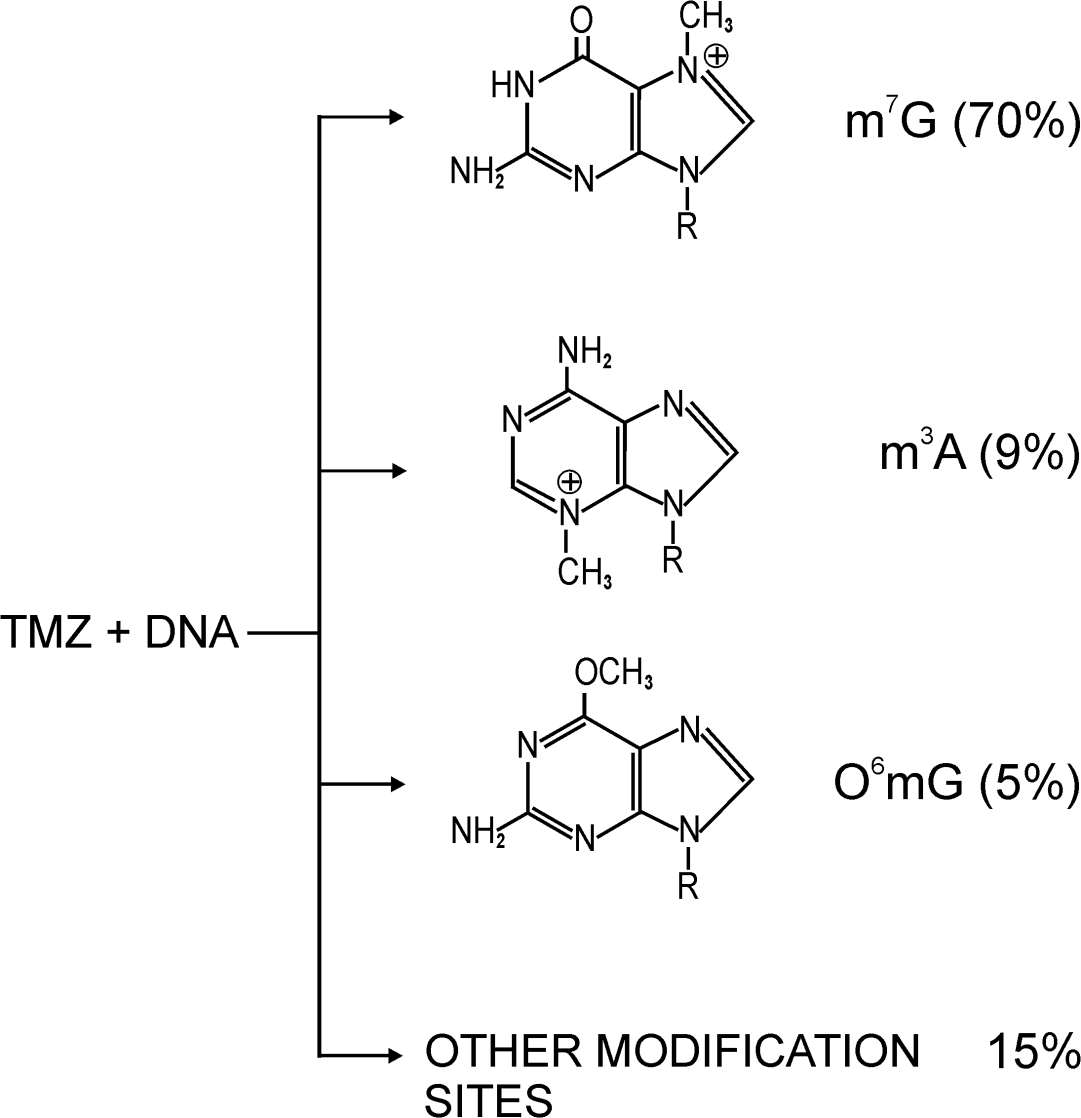
Site specific DNA methylation with temozolomide. The main product of DNA reaction with TMZ is 7- methylguanosine (m^7^G). Methylation of O6 guanosine is supposed to be the main mechanism of TMZ action, but takes place only in 5%.

The prodrug temozolomide (4-methyl-5-oxo-2,3,4,6,8-pentazabicyclo [4.3.0] nona-2,7,9-triene-9-carboxamide with a molecular weight of 194.15), is an imidazole derivative. This is the second-generation alkylating chemotherapy agent developed in the 1980s as part of a rational drug development initiative. Because TMZ is lipophilic, it efficiently crosses the blood–brain barrier and is bioavailable to the CNS. It is stable at acidic pH (< 5), but at neutral and alkaline pH (>7) values rapidly hydrolyses to the active 5-(3-methyltriazen-1-yl) imidazole-4-carboxamide (MTIC) intermediate. In situ formed methyldiazonium ion, is the active compound that transfers the methyl group to DNA bases [10,11]. About 70% of adducts are formed at the N7 position of guanine (m^7^G) and 9% at the N3 position of adenine (m^3^A) (Fig 1). These modified DNA components can be repaired by the base-excision repair (BER) mechanism [12]. The efficient repair minimizes the effect of these lesions. However, if BER is disrupted, these adducts become a highly cytotoxic. BER disruption is able to bypass other TMZ-resistance factors such as over expression of O6 methylguanosine methyltransferases (MGMT) and mismatch repair defects. An approach to enhance TMZ cytotoxicity is to inhibit BER, so that noncytotoxic adducts, i.e. m^7^G and m^3^A, become cytotoxic. Interestingly, only ca. 5% of the methylation reaction mediated by TMZ results with O6 methylguanosine. Despite its low yield, this pathway is currently recognized as the main mechanism of the drug action. The protective effect of MGMT activity in tumour tissue is connected with resistance to TMZ drug. MGMT rapidly reverses modification at O6 position of guanosine, removes the methyl group added by TMZ and reduces the cytotoxic effects of its action [13,14]. It is known that silencing of MGMT gene by the methylation of the cytosine (m^5^C) residue (but not m^7^G, m^3^A or O^6^mG) within the promoter region results in decrease of the enzyme expression in tumour cells. It is well known that cytosine methylation is a common mechanism for inactivating (silencing) tumour suppression genes during malignant progression [12]. It is not clear why methylation of the O6 position of guanosine which represent only a small fraction of the total DNA lesions induced by TMZ (Fig 1), is thought to be the major player of the drug cytotoxic action [11,15]. Methylation of guanine at the O6 results in “mismatch” incorporation of thymidine instead of cytosine, and that error is recognized by the mismatch repair (MMR) enzyme system that attempts to excise thymidine [16]. Since guanine methylation persists, a series of futile replication and repair cycles ensue, ultimately resulting in apoptotic cell death. Because the methylated guanine residues can be repaired, the cells can escape TMZ-induced cell death. Therefore to exert the cytotoxic action of TMZ, a cell needs an intact MMR system and deficient or low MGMT levels. Conversely, a deficient MMR system coupled with high MGMT expression levels mediates the TMZ resistance.

Taking into account a low abundance of O6 methylguanosine and resistance of cancer cells to TMZ, we have asked about the effective mechanism of TMZ action on glioma cells. Instead of DNA methylation adducts, we analyzed changes at the level of m^5^C in DNA of cancer cells after TMZ treatment depending on time and concentration of the drug.

## Materials and Methods

### Cell Lines and Their Treatment with TMZ

Human glioma cell lines (U138MG, U118MG, T98G), HeLa (cervical cancer cells) from human, and glioma C6 cell lines of rat were obtained from ATCC (American Tissue Culture Collection, Rockville, MD, USA). HaCaT cell line (human heart) was from CLS (Cell Line Service GmbH), but temozolomide (TMZ) was from Sigma-Aldrich.

TMZ was freshly dissolved in dimethyl sulfoxide (DMSO) at a concentration of 0.103 M. This stock solution was added directly to culture medium (cells with 90–95% confluence) according to designed concentration. Cells seeded at a density of 5x10^5^ cells per well in 6-well plates containing 2 ml RPMI 1640 or DMEM (Sigma) supplemented with 10% FCS (Sigma) and standard antibiotics **(**penicillin 100 U/ml and streptomycin 100 μg/ml), were grown at 37°C under 5% CO_2_ atmosphere. After 24 h cells were washed with phosphate-buffered saline (PBS, Sigma), placed in fresh medium and treated with TMZ at different concentrations (100, 250, 500 and 1000 μM) for 3, 12, 24 and 48 h. For control the cells were treated with H_2_O or DMSO only. After 3–48 h of TMZ treatment, the cells were washed with PBS, trypsinized and collected by centrifugation at 12000 rpm for 10 min. The cellular pellets were quickly frozen and stored at—20°C for DNA isolation.

### Isolation of DNA

DNA from tissue samples was extracted with Genomic Mini kit (A&A Biotechnology, Gdańsk, Poland). Shortly, tissue samples were incubated with proteinase K first and then with RNase A. After centrifugation (15000 rpm for 3 min), the supernatant was applied to mini column and DNA bound to the column was eluted with Tris-buffer pH 8.5 and stored at -20°C for further analysis.

### DNA Hydrolysis, Labeling and TLC Chromatography

**Fig 2:**
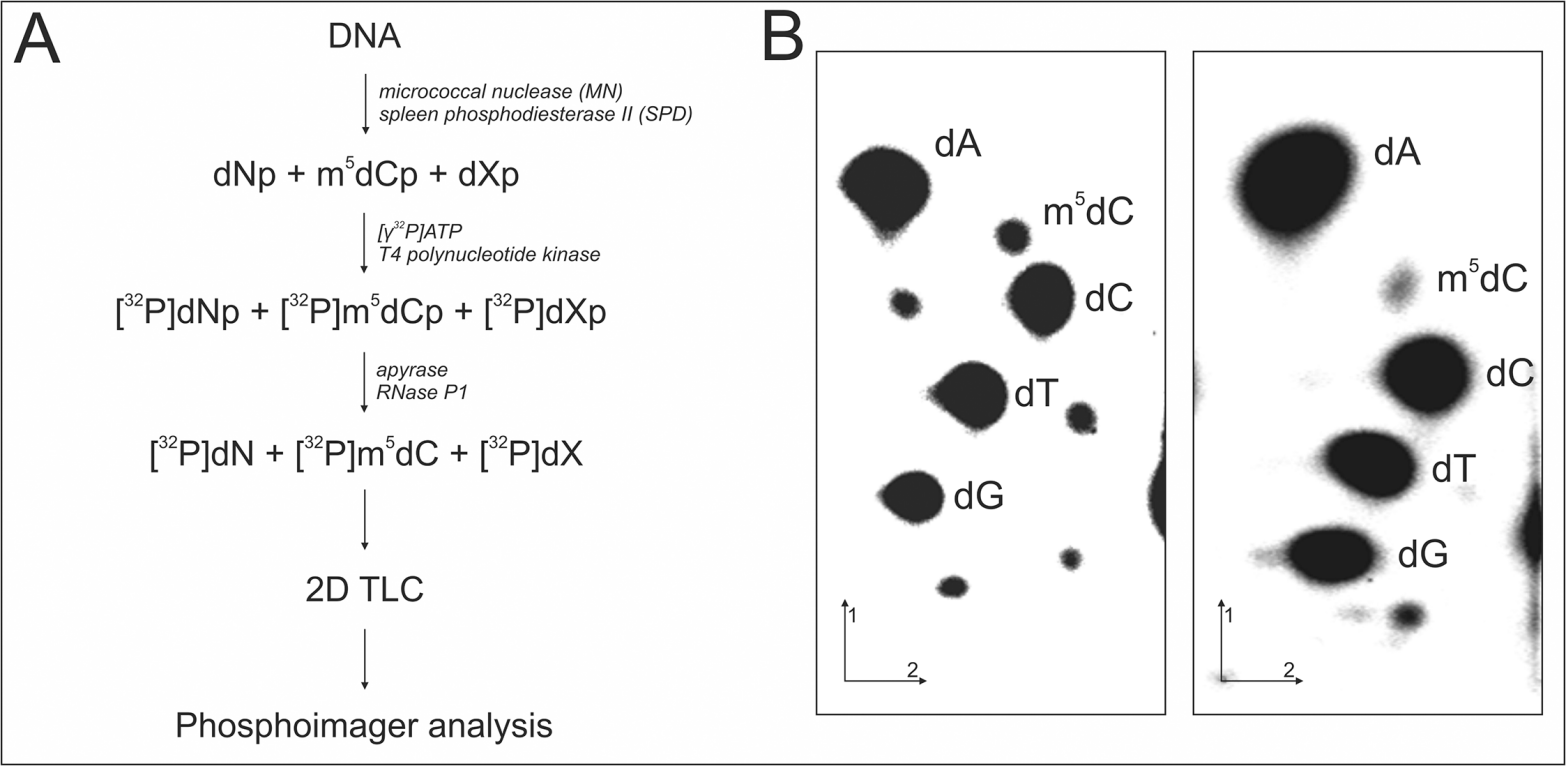
Analysis of m^5^C in DNA. A. Flow chart of the analysis of m^5^C in genomic DNA. Isolated DNA was hydrolyzed to 3’mononucleotides (A—adenosine, G—guanosine, C—cytidine, T–thymidyne and X–other modifications). Furthermore they were labelled with [γ-^32^P], dephosphorylated of 3’ phosphate and separated with TLC in two dimensions. The chromatogram was evaluated with phosphoimager. B. Two dimensional cellulose thin layer chromatography (TLC) analysis of [5’-^32^P] labelled deoxynucleotides obtained by enzymatic hydrolysis of DNA from different types of cells. Pure DNA was isolated from non treated HeLa cells (left) and treated with TMZ (right) for 48 h.

DNA (dried, 1μg) was dissolved in succinate buffer (pH 6.0) containing 10 mM CaCl_2_ and digested with 0.001 units of spleen phosphodiesterase II and 0.02 units of micrococcal nuclease in 3.5 μl total volume for 5 h at 37°C. 0.17 μg of DNA digest was labeled with 1μCi [γ-^32^P]ATP (6000 Ci/mmol, Hartmann Analytic GmbH) and 1.5 units of T4 polynucleotide kinase (USB, UK) in 3 μl of 10 mM bicine-NaOH pH 9.7 buffer containing 10 mM MgCl_2_, 10 mM DTT, and 1 mM spermidine. After 0.5 h at 37°C 3 μl of apyrase (10 units/ml) in the same buffer were added and incubated for another 0.5 h. The 3’nucleotide phosphate was cleaved off with 0.2 μg RNase P1 in 500 mM ammonium acetate buffer, pH 4.5. Identification of [γ-^32^P]m^5^dC was performed with two-dimensional thin-layer chromatography (TLC) on cellulose plates (Merck, Darmstadt, Germany) using solvent system: isobutyric acid:NH_4_OH:H_2_O (66:1:17 v/v) in the first dimension and 0.2 M sodium phosphate (pH 6.8)-ammonium sulfate-n-propyl alcohol (100 ml/60 g/2 ml) in the second dimension. Radioactivity was subsequently measured using a Fluoro Image Analyzer FLA-5100 with Multi Gauge 3.0 software (FujiFilm). Each analysis was repeated four times (Fig 2). For precise calculations we evaluated spots corresponding not only to m^5^dC, but also to products of its degradation as dC (cytosine) and dT (thymine). Amount of m^5^C was calculated as R = [(m^5^dC/m^5^dC+dC+dT)]*100 [17,18].

The data obtained with the post-labeling and TLC methods were identical to those get with ELISA assay [19].

### Cell Cycle Analysis by Flow Cytometry

5 x 10^5^ cells were seeded onto 6-well cell culture plates and incubated for 12 and 48h with 100–1000 μM temozolomide. Control cells were incubated in a fully supplemented growth medium with and without DMSO (17.6 μl). At the end of incubation time, the cells were fixed with 80% ethanol. Then, the cells were harvested and centrifuged at 200 x g for 5 min, and the cell culture medium was removed. The pellet was washed twice with 1ml of PBS, centrifuged as previously and resuspended in a 100 μl of PBS. Then 1 ml of ice-cold 80% ethanol was added and incubated for 2 h in 4°C and stored at -20°C. Before the cell cycle analysis by flow cytometry was done, fixed cells were stained with propidium iode (PI). After centrifugation (200 x g for 5 min) cells were washed twice with 1 ml of PBS and spinned down. The pellet was incubated in a 100 μl of PBS containing 1% of FBS, PI (5 μg) and RNase A (20 μg) for 30 min, 37°C in the dark. The PI fluorescence was measured by FACS calibur flow cytometer (Becton Dickinson). Data were analyzed by FlowJo software [20].

### Statistical analysis

STATISTICA software, 1998 edition (StatSoft Polska, 1995–2008) was used for statistical analyses of all data as in previous our studies [21]. Standard deviations were indicated as errors bars on graphs.

## Results

**Fig 3:**
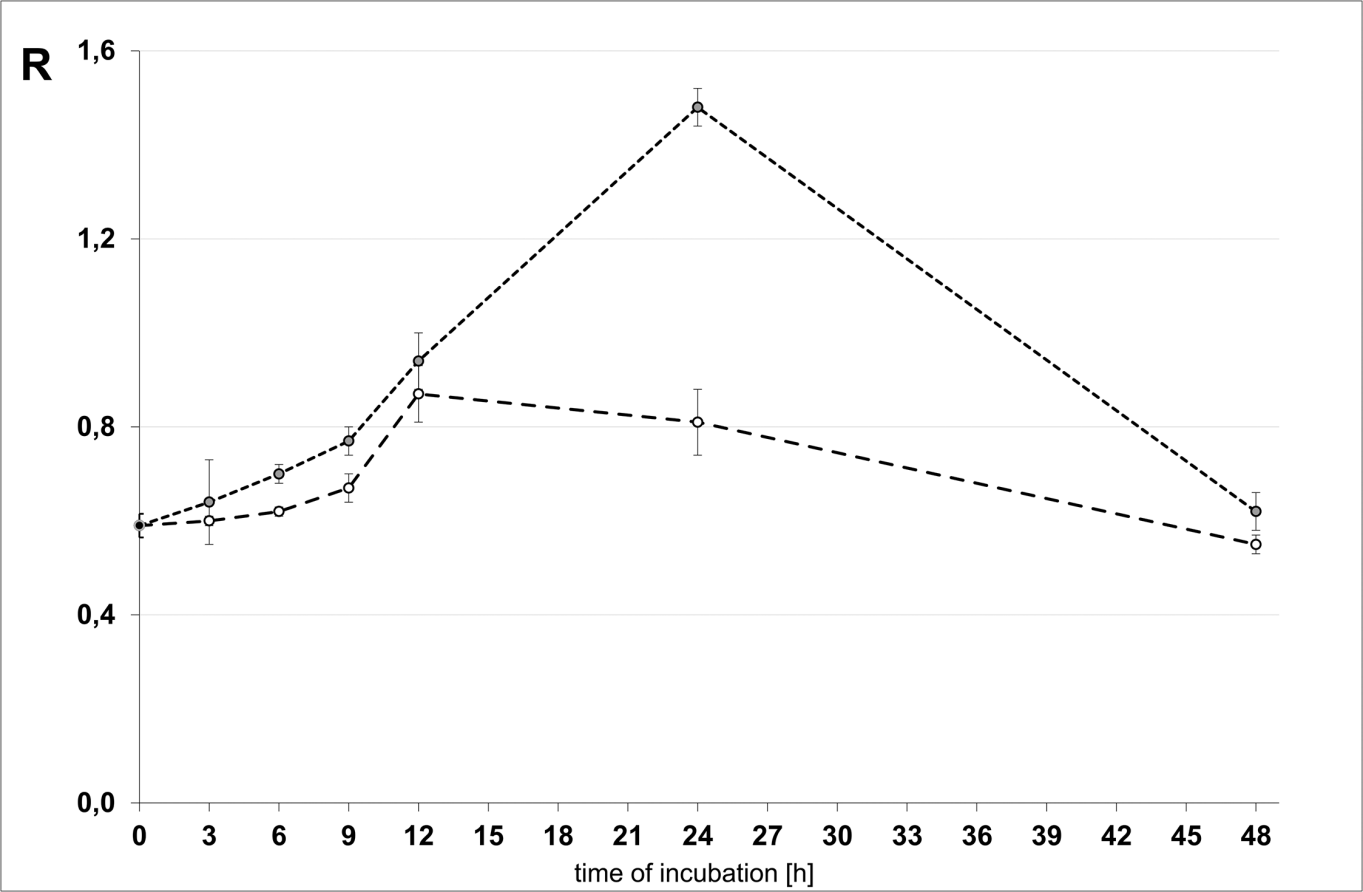
Time effect of TMZ on the DNA methylation status. U118 cell line treated with 250 μM (empty circle) and 500 μM (gray circle) of TMZ at 3, 6, 9, 12, 24 and 48 h. DNA not treated with temozolomide was used as a control (black circle). The contents of m^5^C is indicated on the Y axis. The highest content of m^5^C in DNA was observed after incubation of the cells 24 h with 500 μM of TMZ.

**Fig 4:**
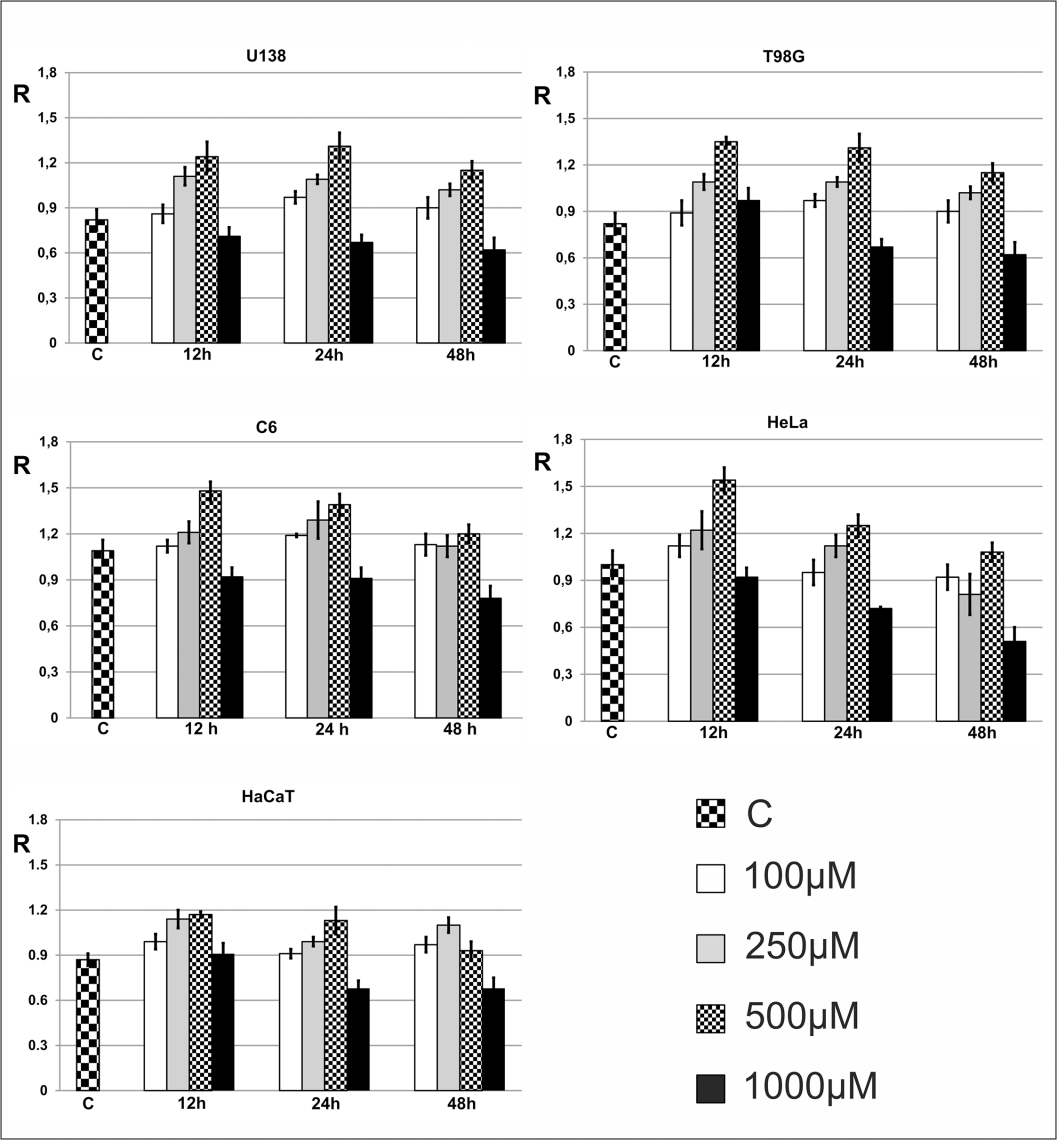
The effect of TMZ on 5 methylcytosine (m^5^C) contents in DNA of glioma. (U138, T98G, C6) and HeLa cells at 3, 12, 24 and 48 h. HaCaT cell line was used as the reference. C stands for control cells treated with DMSO only.

We analysed epigenetic changes in DNA (analysis of m^5^C) of six types of cells: glioma C6 (rat), U118MG (human), U138MG (human), T98G (human), HeLa (human) and HaCaT (human) after treatment with 100–1000 μM of TMZ during 3–48 h. Interestingly, we noticed that U118 cell lines respond to TMZ depends on concentrations and time of reaction. For a short time of TMZ treatment the amount of m^5^C in DNA significantly increases (Fig 3). The amount of m^5^C (R) reaches the highest level at 500 μM TMZ and after 24 h (Fig 3). Prolonging the time of treatment results in decreasing of the level of m^5^C, what clearly suggests DNA demethylation. The highest decrease of R one can observe at the highest TMZ concentration (1000 μM) and 48 h (Fig 4). The final amount of m^5^C is similar to that of non-treated cells. For lower concentration of TMZ (100–500 μM) one can see two effects. The first one, at a short treatment time, up to 24 h is an increase of m^5^C contents in DNA (hypermethylation) and then with longer time, the decrease of methylation was observed. The same effects are noticed for other glioma cell lines as U138, T98G and C6 (Fig 4). In the non-glioma HeLa cancer cell line DNA demethylation rate correlates with TMZ concentrations (Fig 4). The smallest changes of DNA methylation in normal cell line (HaCaT) changes slightly. It is clear that TMZ induces a strong oxidative stress and the decrease of m^5^C in DNA (e.g. demethylation through m^5^C oxidation). The data in Fig 4 clearly suggest that TMZ is more effective (toxic) for cancer than for normal cells.

**Fig 5:**
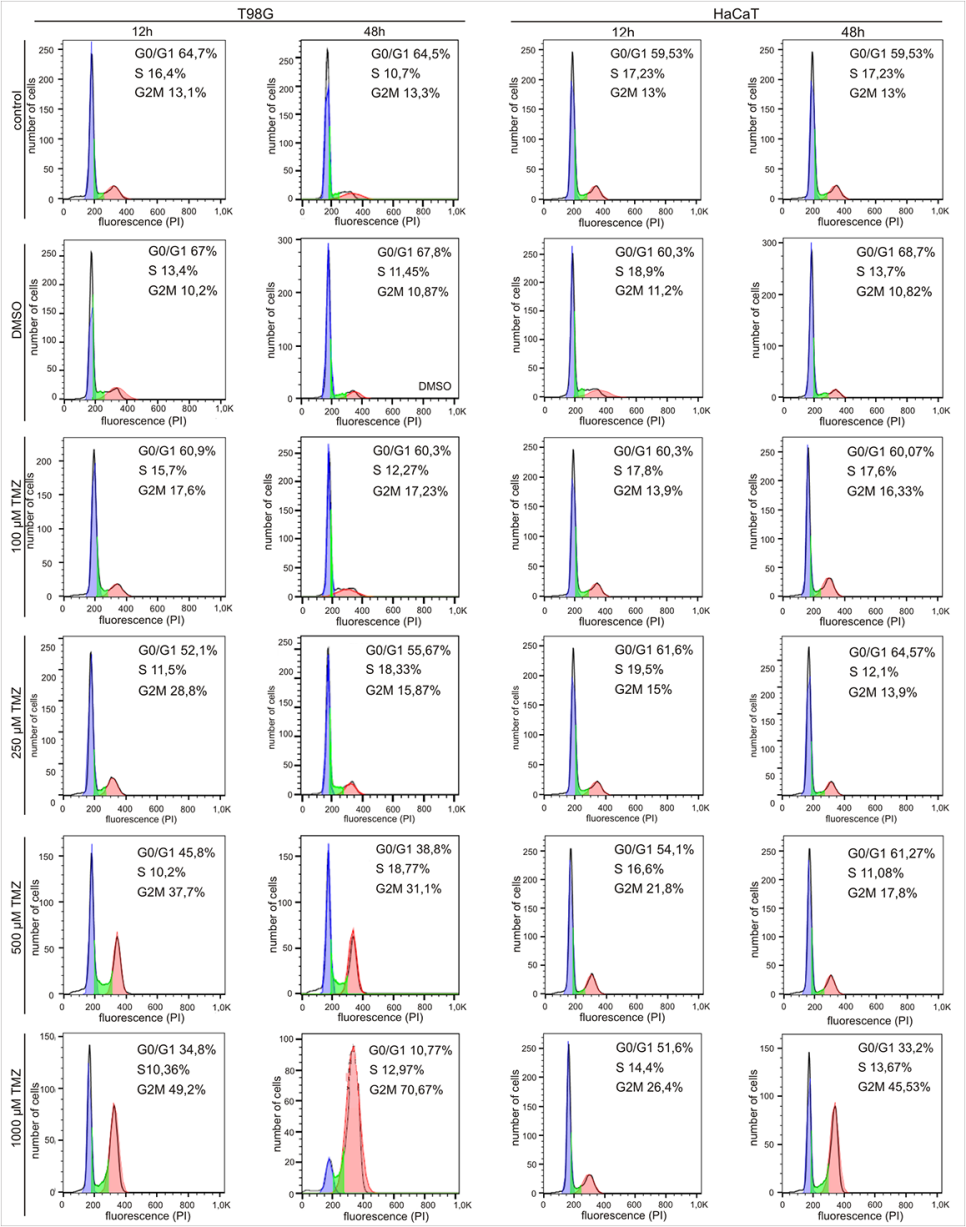
The effect of TMZ on the cell cycle. Cell cycle phases distribution in human glioblastoma (T98G) versus non cancer (HaCaT) cell lines after 12 and 48h temozolomide (TMZ) treatment was carried out using FACS calibur flow cytometer by DNA staining with propidium iode. TMZ treatment resulted in G2M phase arrest in both tested cell lines in a dose dependent manner.

The effects of TMZ on normal HaCaT and T98G glioma cell proliferation were evaluated by quantification with propidium iode (PI). The cell cycle of untreated cells was characterized by long and well-defined G0/G1 peak, with a slightly prominent G2/M and a relatively low S fraction. We noticed that the effect of TMZ on HaCaT cells is weak and smaller than on glioma cells (Fig 5). TMZ treatment of glioma cells at concentrations higher than 500μM and for 12 h induces minor, but during 48 h incubation large changes were observed at the concentration of 1000 μM (Table 1). Treatment with TMZ reduces the amount of cells in sub G0/G1, but the percentage of cells in S and G2/M significantly increases (Fig 5 and Table 1). These changes were more evident for cells incubated for 48 h with TMZ. In the presence of 500 and 1000 μM of TMZ, the percentage of cells in the sub G0/G1 phase decreased to 25.7% and 53.73%, but amount of cells in G2/M phase increased up to 17.8% and 57.37% respectively (Table 1). The changes are bigger for T98G than for HaCaT cells (Table 1).

**Table 1:** The effect of TMZ on the HaCaT and T98G cell cycle lines. The percentage of cells in each phase of the cell cycle in cancer (T98G) versus non cancer (HaCaT) cell line after the drug treatment. The highest concentrations of TMZ (500 μM and 1000 μM) affected the most cell cycle phase distribution. The results are expressed as a mean ± SD. All experiments were done in triplicate.

TMZ Concentration [μM]	HaCaT			T98G		
	G1 (%)	S (%)	G2/M (%)	G1 (%)	S (%)	G2/M (%)
	**12 h**					
**0**	**59.5**	**17.2**	**13.0**	**64.7**	**16.4**	**13.1**
**100**	**60.3**	**17.8**	**13.9**	**60.9**	**15.7**	**17.6**
**250**	**61.6**	**19.5**	**15.0**	**52.1**	**11.5**	**28.8**
**500**	**54.1**	**16.6**	**21.8**	**45.8**	**10.2**	**37.7**
**1000**	**51.6**	**14.4**	**26.4**	**34.8**	**10.4**	**49.2**
	**48 h**					
**0**	**59.5**	**17.2**	**13.0**	**64.5**	**10.7**	**13.3**
**100**	**60.1**	**17.6**	**16.3**	**60.3**	**12.3**	**17.2**
**250**	**64.6**	**12.1**	**13.9**	**55.7**	**18.3**	**15.9**
**500**	**61.3**	**11.1**	**17.8**	**38.8**	**18.8**	**31.1**
**1000**	**33.2**	**13.7**	**45.5**	**10.8**	**13.0**	**70.7**

**Fig 6:**
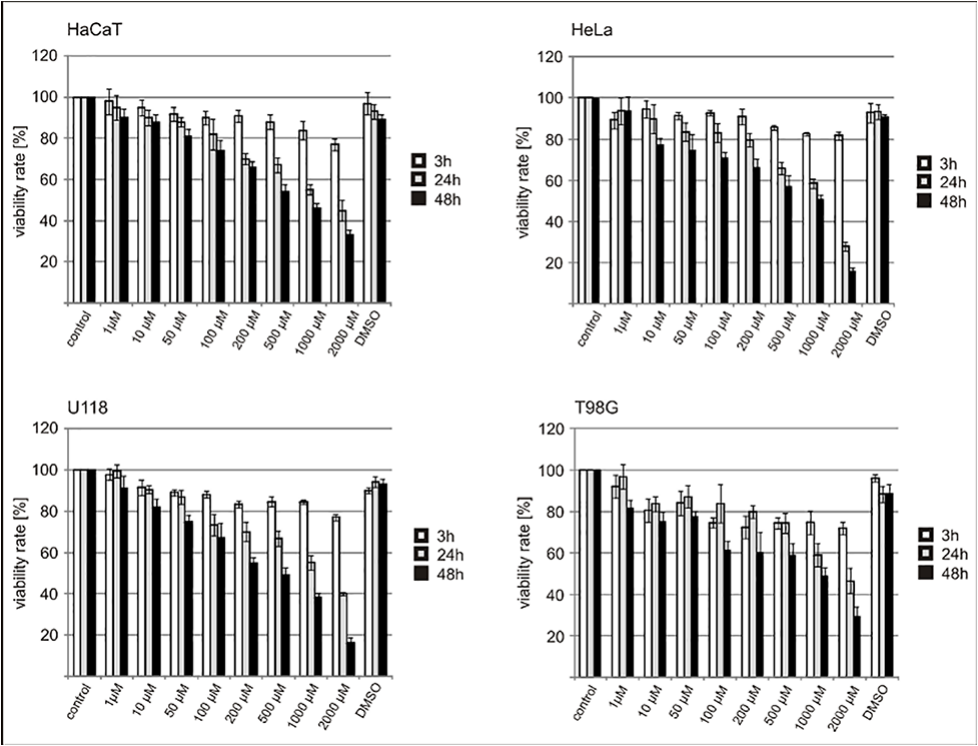
The effect on cell viability after temozolomide treatment estimated by MTT assay. Normal (HaCaT) and tumor cells (HeLa, T98G and U118)were treated with different TMZ concentrations (1–2000 μM) for 3-48h. C- control cells cultured without TMZ; DMSO- cells treated with the highest DMSO concentration used in the experiments. The results are expressed as a mean ± SD.

Finally we carried out cytotoxicity (MTT) assay. It was shown that TMZ treatment in the range of 1–2000 μM trigger cell death apoptosis independent (Fig 6).

## Discussion

Resistance to temozolomide via DNA repair mechanism based on O6 methylguanosine methyltransferases remains a significant issue for the successful treatment of patients with malignant glioma. An expression of MGMT in tumour cells may determine response to TMZ and silencing of MGMT gene by promoter methylation plays an important role in regulation of MGMT activity in gliomas. Based on MGMT promoter methylation modulation, some approaches to overcome MGMT mediated resistance have been proposed [11]. Although TMZ does not cure GBM, it has been shown that its anti glioma activity prolongs overall survival [12]. To explore the molecular mechanism underlying the action of TMZ, we have looked at the other level of genetic information and analysed global changes of 5-methylcytosine (m^5^C) the main epigenetic modification of DNA in glioma cell lines. The DNA methylation profile itself is not static and is subjected to dynamic changes induced by aging, environmental, nutritional and pathogenic factors, viruses and many others [20].

It is known that DNA methylation (m^5^C) can function as a "switch" to activate or repress a gene transcription, providing an important mechanism for tissue specific and developmentally regulated genetic processes [22]. The C5 of cytosine residue can be activated only with specific DNA methyltransferases (DNMT), which catalyse the side specific DNA methylation using S-adenosylmethionine (SAM) as the substrate. In normal cells, the level of m^5^C is controlled by enzymatic DNA methylation–demethylation processes. The amount of m^5^C in the genomic DNA can be measured by a wide range of methods designed to yield quantitative and qualitative information on genomic DNA methylation [23,24]. Taking into account the regulatory role of m^5^C, together with its chemical reactivity and capacity as a mutational hotspot [25], we studied the genomic (global) amount of m^5^C in DNA of different cancer cells treated with TMZ at various conditions to monitor its action on the DNA epigenetic mark. The amount of m^5^C expressed as R coefficient is a good diagnostic biomarker for tumorgenesis processes and proved to be useful as a probe for various diseases and aging [18,24,26]. From m^5^C content analysis of TMZ effect on cancer cells two questions appeared. The first one is how changes and particularly an increase in amount of the epigenetic mark can be explained. Taking into account that TMZ is not able to react directly with the cytosine C5 carbon and it is not the substrate for DNA methyltransferases, the only reasonable conclusion seems to be that temozolomide stimulates or activates the enzyme responsible for specific m^5^C formation in DNA e.g. DNA methyltransferase. As a consequence of that, one can see the methylation of the promoter region of gene of some proteins and their silencing. On the other hand, TMZ as well as other drugs administered for longer time and at high concentrations induces significant cellular stress [27]. Indeed, TMZ induced hypomethylation suggests global stress (oxidative damage) to the cell and particularly to all DNA constituents including m^5^C. Such a random and global removal of the epigenetic marker being the silencing signal, leads directly to the activation of gene expression of pathology related proteins. This conclusion is supported by increasing number of cells and the arrest of glioma cells at G2/M phase due to numerous modifications that occur to DNA bases to DNA damage.

The second issue is how the observations from global epigenetic analysis fit to clinical data concerning temozolomide administration to patients with glioblastoma. It is known that global hypomethylation is a general property of all human cancers [28]. The same are due to brain tumours. Recently we have shown that the level of m^5^C in DNA of brain tumours decreases as their malignancy increases [26,29]. The level of m^5^C in DNA of glioblastoma cells is the lowest among human cancers. In contrary, brain tumours of WHO grade I and II showed significantly higher amount of m^5^C in DNA. Therefore DNA hypermethylation observed in cells in the first phase of TMZ treatment can be very useful for patient but further demethylation induced with TMZ, promote–errous in gene expression and further malignancy. The lesson here is that small dosages of TMZ given for a shorter time should be considered as more beneficial to patients. Cell toxicity experiments have shown, that TMZ affects cell viability in a dose and time dependent manner. Incubation with TMZ in a concentration range 1 – 2000uM up to 48h resulted in a decrease of cell viability up to ~16% in HeLa and U118 cells. Our observation is consistent with other reports [30–32]. Moreover it has been also shown that TMZ cytotoxicity is not due to induction of apoptosis [31–32].

Finally the obvious question is which mechanism of TMZ action is the functional one? It seems that one based on O6 methylation of guanines (ca. 5%) which implies O6-methylguanine-DNA methyltransferase involvement in acquired resistance of cells to TMZ, has some weak points, particularly a low percentage of methylation of this site. The other refer to global hyper- and hypomethylation which underlines the crucial role of epigenetics in carcinogenesis.

Finally, our data strongly support that TMZ acts mostly through oxidative stress induction and DNA methylation status changes. It is clear that TMZ induces an increase in m^5^C content in DNA in the first stage of treatment and decrease in m^5^C amount in DNA in the second step. Therefore it is obvious that hypermethylation is a result of higher DNA methyltransferases’ activity induced with TMZ and that process down regulate the expression of some genes potentially involved in carcinogenesis. One can consider that as a positive effect of TMZ. On the other hand hypomethylation is an effect of oxidative stress and results in development or progression of cancer. Finally TMZ administration with low doses of the drug and at short times should be considered as an optimal therapy.

## Conclusions

Our results of studies on TMZ treatment suggest the new mechanism of its action. It works through epigenetic marker (m^5^C) modulation in cancer cells. A high TMZ concentration in the short time induced an increase of m^5^C content in DNA, but at low TMZ concentration and longer time hypomethylation is observed in whole range of the drug concentrations. Therefore administration with low doses of TMZ and short time should be considered as optimal therapy.
